# Supplementary material for: Higher Plasma Creatinine Is Associated with an Increased Risk of Death in Patients with Non-Metastatic Rectal but Not Colon Cancer: Results from an International Cohort Consortium
Source: Cancers (Basel). 2023 Jun 28;15(13):3391. doi: 10.3390/cancers15133391 (PMC10340258; doi:10.3390/cancers15133391)
Supplement: Supplementary file 1 [file cancers-15-03391-s001.zip › cancers-2354700-supplementary/Supplementary Table_S3_ Baseline characteristics by cohort.pdf]

Table S3. Baseline characteristics of the total study population of MetaboCCC overall and stratified by cohort

COHORTS	**Total population**	**COLON**	**EnCoRe**	**CORSA**	**ColoCare HD**
**Number of participants**, n (%)	680	192 (28%)	206 (30%)	55 (8%)	227 (33%)
**Age at diagnosis**, years (median, range)	66 (60-73)	67 (62-72)	67 (60-72)	70 (61-73)	65 (56-73)
**Sex**, n (%)					
Male	440 (65%)	110 (57%)	133 (64%)	44 (80%)	152 (67%)
Female	240 (35%)	81 (42%)	73 (36%)	11 (20%)	75 (33%)
**Vital status,** n (%)					
Alive	587 (86%)	167 (87%)	170 (83%)	45 (82%)	205 (90%)
Deceased	93 (14%)	25 (13%)	36 (17%)	10 (18%)	22 (10%)
**Follow-up time,** years (median, range)					
Alive	4.48 (3.02-5.85)	6.06 (5.50-6.57)	4.65 (4.41-5.42)	5.17 (3.95-6.09)	2.21 (1.97-3.04)
Deceased	2.80 (1.1-4.15)	4.42 (3.52-5.66)	2.51 (0.9-3.46)	2.07 (1.16-2.4)	1.44 (0.34-2.16)
**Stage of disease,** n (%)					
I	176 (26%)	56 (29%)	53 (26%)	26 (47%)	41 (18%)
II	215 (32%)	66 (34%)	51 (24%)	15 (27%)	83 (37%)
III	289 (42%)	70 (36%)	102 (50%)	14 (26%)	103 (45%)
**Tumor location[1*],** n (%)					
Colon	394 (58%)	126 (66%)	128 (62%)	37 (71%)	103 (45%)
Rectal	283 (42%)	65 (34%)	79 (38%)	15 (29%)	124 (55%)
**Neo-adjuvant treatment,** n (%)					
Yes	181 (27%)	60 (31%)	60 (29%)	1 (2%)	61 (27%)
No	478 (73%)	132 (69%)	146 (71%)	54 (98%)	166 (73%)
**Surgery,** n (%)**					
Yes	669	192 (100%)	196 (100%)	54 (100%)	227 (100%)
**Adjuvant treatment,** n (%)					
Yes	206 (31%)	45 (24%)	69 (34%)	15 (27%)	77 (36%)
No	457 (69%)	143 (76%)	137 (66%)	40 (73%)	137 (64%)
**Body mass index**					
**Continuous**, kg/m^2 (median, range)	26.6 (13.3-46.00)	25.4 (17.20-40.2)	27.9 (18.2-46.0)	26.2 (20.2-37.1)	26.3 (13.3-39.7)
Underweight, <18.5, n (%)	7 (1%)	3 (2%)	1 (<1%)		3 (1%)

Normal weight, 18.5-24.9, n (%)	215 (32%)	81 (43%)	44 (21%)	14 (30%)	76 (34%)
Overweight, 25-29.9, n (%)	293 (44%)	76 (41%)	93 (45%)	21 (46%)	103 (46%)
Obese, ≥30, n (%)	148 (23%)	27 (14%)	67 (32%)	11 (24%)	43 (19%)
**Height,** cm (median, std)	1.72 (1.66-1.79)	1.73 (1.67-1.79)	1.71 (1.64-1.78)	1.75 (1.66-1.86)	1.72 (1.66-1.79)
**Weight,** kg (median, std)	80 (70-90)	76 (68-85)	80 (72-93)	83 (71-100)	80 (70-88)
**Smoking,** n (%)					
Current	102 (15%)	20 (11%)	27 (13%)	11 (21%)	44 (20%)
Former	341 (52%)	114 (60%)	110 (54%)	18 (35%)	99 (46%)
Never	216 (33%)	56 (29%)	65 (33%)	23 (44%)	72 (34%)
**Alcohol intake in the past year,** n (%)					
Yes	572 (87%)	152 (80%)	164 (82%)	29 (64%)	227 (100%)
No	89 (13%)	37 (20%)	36 (18%)	16 (36%)	

[1]Tumor location is defined as colon (cecum, appendix and ascending colon, hepatic flexure, transverse colon, splenic flexure, descending colon and sigmoid colon) and rectal (rectosigmoid junction and rectum) cancer. *Patients with missing information on study site were excluded from the present study. **Analyses were not adjusted for surgery as all participant received surgery.
