# Supplementary figures and images for: Higher Plasma Creatinine Is Associated with an Increased Risk of Death in Patients with Non-Metastatic Rectal but Not Colon Cancer: Results from an International Cohort Consortium
Source: Cancers (Basel). 2023 Jun 28;15(13):3391. doi: 10.3390/cancers15133391 (PMC10340258; doi:10.3390/cancers15133391)

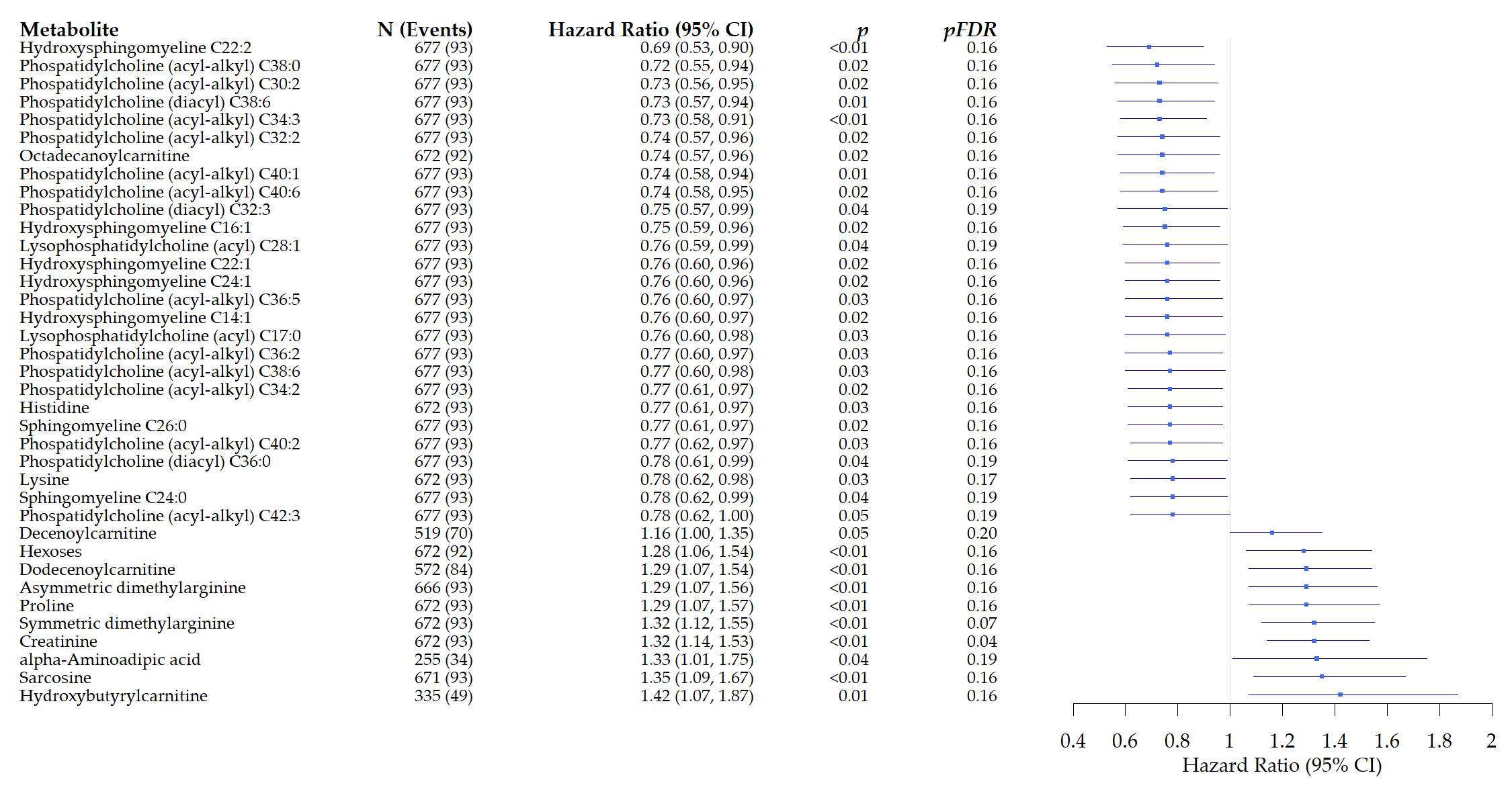

Supplement: Supplementary file 1 [file cancers-15-03391-s001.zip › cancers-2354700-supplementary/Supplementary Figure S1. Associations of metabolites with all-cause mortality in the entire study population.tiff]
